# Exploration of the Implementation of Carbon Neutralization in the Field of Natural Resources under the Background of Sustainable Development—An Overview

**DOI:** 10.3390/ijerph192114109

**Published:** 2022-10-28

**Authors:** Wu Yang, Zhang Min, Mingxing Yang, Jun Yan

**Affiliations:** 1 Faculty of Resources and Environmental Engineering, Guizhou Institute of Technology, Guiyang 550003, China; 2 Engineering Research Center of Carbon Neutrality in Karst Areas, Ministry of Education, Guiyang 550003, China

**Keywords:** soil carbon sink, forest carbon sink, karst carbon sink, carbon sequestration, carbon neutralization, environment

## Abstract

On 15 March 2021, Chinese President Xi Jinping pointed out that “achieving carbon peak and carbon neutrality is a broad and profound economic and social systemic change” and called for “putting energy and resources conservation in the first place”. Natural resources are the material basis, space carrier and energy source of high-quality development. The source of carbon emissions is resource utilization, and carbon reduction and removal also depend on resources. The improvement of carbon sink capacity is inseparable from natural resources. To achieve the goal of “double carbon”, it is necessary to consolidate the carbon sink capacity of the ecosystem, as well as enhancing its carbon sink increment. Among natural resources, forest carbon sinks, soil carbon sinks and karst carbon sinks have significant emission reduction potential and cost advantages, representing important means to deal with climate change. This paper reviews the relevant research results at home and abroad, summarizes the carbon sink estimation, carbon sink potential, carbon sink influencing factors, ecological compensation mechanism and other aspects, analyzes the path selection of establishing carbon sink green development, and puts forward corresponding policies and suggestions, providing a theoretical reference for the achievement of the carbon neutrality goal in the field of natural resources in China.

## 1. Introduction

Striving to achieve carbon peak by 2030 and carbon neutrality by 2060 is a major strategic decision made by China, an inevitable choice to solve the outstanding problems of resource and environmental constraints and realize the sustainable development of the Chinese nation, and a solemn commitment to building a community of human destiny. Achieving carbon peak and carbon neutralization is an extensive and profound economic and social systemic change, which cannot be achieved easily.

One of the prominent problems in promoting carbon peak and carbon neutralization is the contradiction between high carbon emissions and an insufficient carbon sink capacity, as well as the quantitative relationship between carbon sources and carbon sinks. Among them, carbon sources are a systemic problem based on the energy structure, which is related to the path of economic development and the adjustment of the industrial structure, and the core is to reduce the intensity and total amount of carbon emissions; carbon sinks are an indispensable link to achieve carbon neutralization, and negative carbon technology innovation will play a central supporting role. Natural resource work runs through the whole process and all links of the source–sink relationship, which is the key to solving this contradiction [1,2,3].

**Figure 1:**
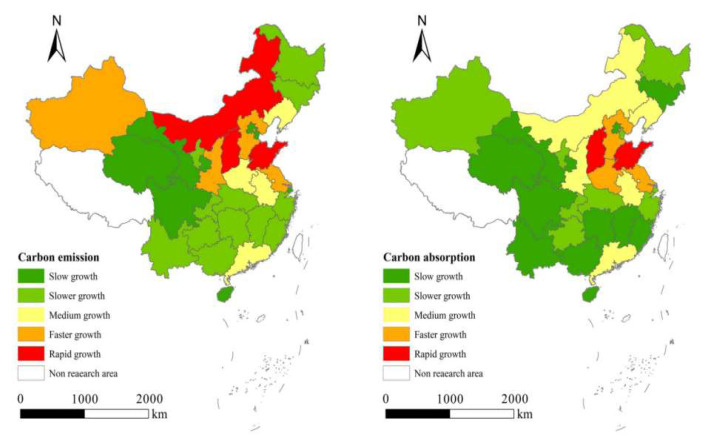
Growth level of carbon emissions and total carbon absorption in provinces from 2003 to 2016 (Huang, H. [7]).

China is expected to emit about 12 billion tons of carbon per year in 2030, and then gradually reduce carbon emissions to 3 billion tons per year by 2060. While ensuring national economic development, this is a “difficult, heavy task, time is tight” work, if only rely on “emission reduction” is difficult to achieve carbon neutrality. Therefore, more and more attention should be paid to the way of “increasing carbon sink”, and the capacity of ecological carbon sink should be tapped to enhance the increment of ecosystem carbon sink. Therefore, it is very important to explore the implementation of carbon neutrality in the field of natural resources (Figure 1).

Natural resources can help reduce carbon emissions by consolidating and enhancing the carbon sink capacity of the ecosystem and the implementation of underground carbon dioxide storage, to provide a strong guarantee for the realization of the goal of double carbon. Enhancing the carbon sink capacity of ecosystems involves the process of absorbing carbon dioxide in the atmosphere through afforestation, vegetation restoration and other measures, but it also emphasizes the balance and maintenance of various ecosystems and their interrelated whole in the global carbon cycle. From this point of view, the focus is on strengthening the overall protection of natural resources and maintaining their carbon sink stocks, rather than substantially increasing the carbon sink increment in the short term [4,5,6,7].

Part of the carbon dioxide emitted by human beings stays in the atmosphere, while the other part is absorbed by terrestrial and marine ecosystems, the latter mainly including forest ecosystems, river and lake wetland ecosystems and farmland ecosystems. Among them, forests are the largest “carbon reservoir” and the most economical “carbon absorber” in terrestrial ecosystems, with a contribution rate of about 80%, and their annual average carbon sequestration can offset 11% of fossil fuel carbon emissions in the same period. According to the research on the increase in the carbon sequestration of terrestrial ecosystems in China in recent decades, 60% of the increase in the carbon sequestration of plantations has come from the increase in forest area. In the future, we should consolidate the achievements of forest carbon sequestration, paying particular attention to the role of the carbon adsorption of forest ecosystems. Wetlands are an important source of carbon sinks in known terrestrial ecosystems, second only to forests, and play an important role in absorbing greenhouse gases from the atmosphere, slowing down global warming and achieving the goal of double carbon. As for farmland ecosystems, their role in carbon sequestration has not been recognized by the United Nations Intergovernmental Panel on Climate Change, and the main direction of enhancing farmland carbon sinks is to enhance soil organic carbon storage through actions to improve the quality of cultivated land and the application of sink-enhancing agricultural technologies [8,9].

**Figure 2:**
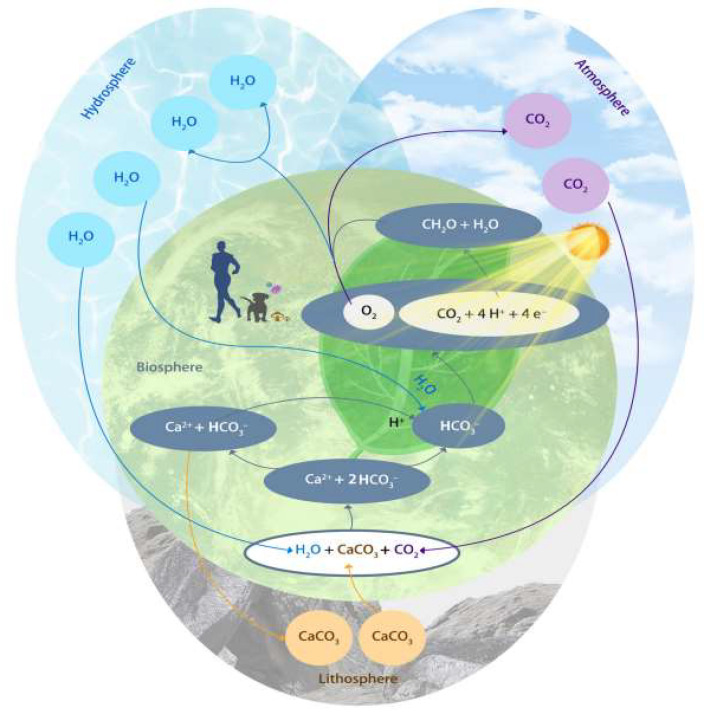
Karstification–photosynthesis coupling processes and their role in the water–carbon balance in nature (Yanyou Wu [12]).

It is worth noting that the weathering of carbonate rocks is also considered to be part of the terrestrial weathering process. Since 2016, through the project of “Comprehensive Environmental Geological Survey of Carbon Cycle in Karst Basins of the Yangtze River, Pearl River and Yellow River” undertaken by the Chinese Karst Institute, the conceptual model of the karst carbon cycle at the basin scale has been established based on the research and analysis of historical data, and the migration and transformation process of the carbon element from inorganic carbon to organic carbon and then to inert organic carbon in karst basins has been clarified. The long-term dynamic monitoring results also show that 70% to 80% of the total carbon dioxide consumed by the atmosphere and soil by carbonate weathering in the whole basin is stable. This strongly responds to the international query about “the stability of carbon sink in chemical weathering of carbonate rocks” and thus draws the conclusion that the karst carbon cycle can produce carbon sinks on a short time scale, which provides a basis for carbon sink flux calculation and model research. Under the unified deployment of the China Geological Survey, the Karst Institute has carried out investigations and monitoring in the Yangtze River, Pearl River and Yellow River Basins in recent years. Based on the monitoring data of nine major river basins, the estimation results show that the carbon sink flux produced by the weathering and dissolution of carbonate rocks in China ranges from 0.3 billion tons per year to 72 million tons per year, with an average of 51 million tons per year. “The monitoring data has been used by the United Nations Intergovernmental Panel on Climate Change (IPCC)”, being adopted in its fifth report. Combined with long-term station data and climate change trends, the total annual carbon sink flux of terrestrial vegetation in China from 1981 to 2000 was 101 million tons. Therefore, in China, the karst carbon sink flux accounts for about 50% of the terrestrial vegetation carbon sink flux (Figure 2) [10,11,12].

Although there are many types of carbon sinks in natural resource systems, what is the potential of their carbon sinks? How to help continuously consolidate and enhance carbon sink capacity from the policy level? How should its market economy potential and operation mode be carried out? These are all unsolved problems.In this research, we continue the massive literature reorganization and analysis work, in view of natural ecosystems (soil, forest and karst), analyze the characteristics of various carbon sinks, determine the contribution rate and economic value of various carbon sinks in the ecosystem to achieve the goal of carbon neutrality, which are closely linked to the requirements of the national “double carbon” strategy, and put forward some ideas and suggestions for natural resources to help achieve the goal of carbon neutrality. Therefore, this study provides scientific and technological support for the formulation of ecosystem carbon sequestration management strategies, and has strong policy guidance and applicability.

## 2. Literature Review of Soil Carbon Sequestration

**Figure 3:**
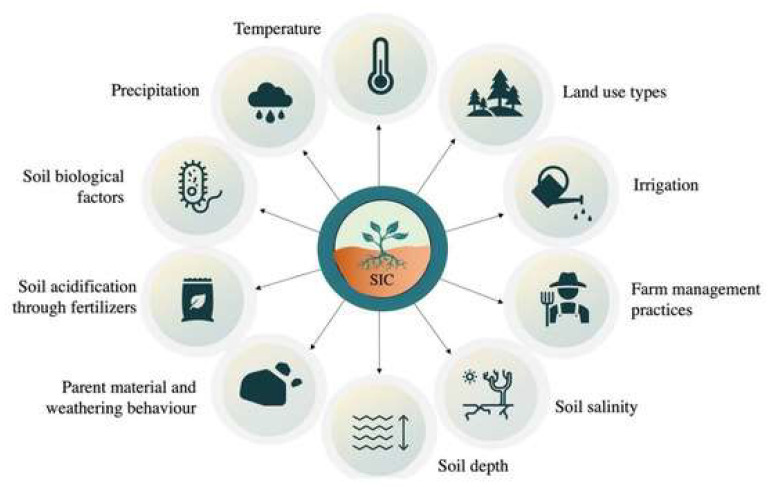
Natural and anthropogenic factors influencing the formation of soil inorganic carbon in dryland soils (Naorem, A [18]).

Among natural resources, soil occupies an important position. According to the report of the Food and Agriculture Organization of the United Nations (FAO), agriculture has become the second largest source of greenhouse gases, but it also has a huge capacity to sequester carbon and reduce emissions. Therefore, reasonable agricultural management measures can be taken to reduce the release of carbon dioxide from soil or enhance the ability of soil carbon sequestration, increase agricultural production, increase soil carbon sequestration and improve the ecological environment. It is urgent for China to develop carbon sink agriculture. China is a large country with significant agricultural greenhouse gas emissions. Under the background of global warming, the sustainable development of agriculture in China is facing severe challenges. Agricultural soil carbon sinks in China have significant potential to reduce emissions, representing an important means to cope with climate change. Therefore, it is very important to deeply understand the current situation of agricultural carbon sinks and carbon sources in China, as well as their spatiotemporal evolution over long periods of time, in order to guide the next step of agricultural modernization and low carbon (Figure 3) [12,13,14,15,16,17,18].

### 2.1. Connotation and Influencing Factors of Soil Carbon Sequestration

As for the connotation of soil carbon sinks, relevant organizations and professionals in the scientific community have offered different definitions. According to the 2006 IPCC Guidelines for National Greenhouse Gas Inventories, soil carbon sinks include organic carbon sinks and inorganic carbon sinks. The former are characterized by the decomposition of plant organic matter into organic carbon in mineral soils, while the latter are characterized by the transformation of atmospheric carbon dioxide into primary or secondary minerals in soils. Soil carbon sequestration is defined by the Soil Society of America as the direct or indirect storage of atmospheric carbon dioxide in soil in the form of stable solids, including the direct conversion of carbon dioxide into soil inorganic substances such as calcium or magnesium carbonate, or the indirect conversion of atmospheric carbon dioxide into plant energy through plant photosynthesis, which is fixed as soil organic carbon in the process of decomposition. Cameron Hepburn of Oxford University defines soil carbon sequestration as the process of increasing the soil organic carbon content through various land management practices. Professor Pan Genxing of Nanjing Agricultural University believes that the process linked to soil carbon sinks is the interception and transformation of atmospheric carbon dioxide into soil solid carbon components, which are realized in the form of soil organic carbon or soil inorganic carbon, indicating the overall performance of the sink effect of soil on atmospheric carbon dioxide.

**Figure 4:**
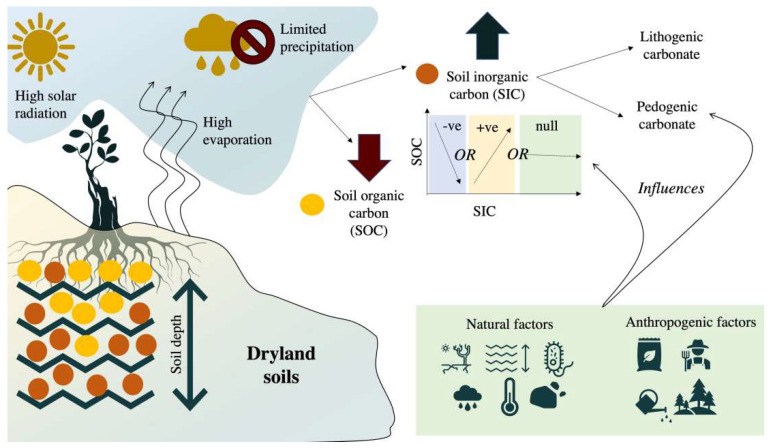
Mechanism of SIC formation in soil (Naorem, A [18]).

From the perspectives of intergovernmental organizations, academic organizations and scholars, the connotation of soil carbon sinks can be understood at two levels. Firstly, soil carbon sinks have two forms: soil organic carbon sinks and soil inorganic carbon sinks. Secondly, the focus of current soil carbon sink research and management practice is soil organic carbon sinks, because the renewal time of inorganic carbon pools is longer, and the active exchange with atmospheric components is mainly soil organic carbon. The soil organic carbon cycle includes two links: the “carbon input” of vegetation fixing carbon dioxide in the atmosphere, and the “carbon output” of microbial decomposition of soil organic carbon. Soil inorganic carbon is the main form of soil carbon pools in arid and semi-arid regions. The mechanisms of soil absorbing carbon dioxide include atmospheric transport, carbonate dissolution and infiltration of soil water in the vadose zone, while the reverse process releases carbon dioxide and forms an inorganic carbon cycle. Many natural and human factors affect the process of the soil carbon cycle together, and the impact of human factors on the soil carbon cycle and carbon storage is far greater than that of natural factors. In terms of natural factors, the changes in the soil carbon pool are affected by a variety of physical and biological factors, such as climate, soil structure and chemical, physical and biological properties, vegetation types and microbial physiological and biochemical processes, and there are interactions among these factors. In terms of human factors, different land cover and land use patterns lead to great differences in the soil carbon sink capacity. Generally, the soil carbon sink capacity of different ecosystems from large to small is forest > grassland > wetland > farmland > unused land and construction land. When human activities cause land use/land cover change (LUCC), on the one hand, this directly changes the ecosystem type, thereby affecting the net primary productivity of the ecosystem and the input of soil organic carbon; on the other hand, this indirectly changes the biological, physical and chemical properties of the soil, thereby affecting the soil respiration and the intensity of soil carbon output(Figure 4) [19,20,21,22].

### 2.2. Soil Carbon Sequestration Potential and Economy

Meta-analysis, soil survey data subtraction and process modeling are three main methods to estimate the change rate of the soil carbon pool at the global and national scales. Meta-analysis mainly uses the relevant data in the existing literature to calculate the change rate of the soil carbon pool, and soil survey data subtraction involves calculating the change rate directly by subtracting the measured data of two soil carbon pool surveys. Process simulation is based on climate, soil physical and chemical properties, agricultural and forestry management measures and other factors to build a mechanism model to estimate the rate of change in the soil carbon pool (common models are the DNDC model, the Agro-C model, etc.). The United Nations Framework Convention on Climate Change (UNFCCC) requires all parties to use comparable methodologies agreed upon by the Conference of the Parties. Therefore, the Intergovernmental Panel on Climate Change (IPCC) began to study and compile the national greenhouse gas emission inventory guidelines in 1995 and has published four versions of the inventory guidelines, the latest of which are the IPCC 2006 Guidelines for National Greenhouse Gas Inventories 2019 Revision. According to the relevant results of the IPCC, there are three main accounting methods for soil carbon sinks [23,24,25,26,27,28]:

(1) The default method, with default values for emission/removal factors and parameters from IPCC-1996-LUCF or IPCC-GPG-LULUCF;
(2) Inclusion of country-specific data, and use of national activity data and emission/removal factors or parameters with higher resolution;
(3) Advanced estimation systems that use measurement and/or modeling methods to improve estimates of carbon emissions and removals.

Many scholars have applied the above methods to study the potential of soil carbon sequestration around the world and in China. Due to differences in data sources and specific methodologies, the estimation results are quite different, but they all agree that soil carbon sequestration has great potential. Many scholars have estimated the technical potential of global soil organic carbon sequestration, and the results show that the potential of global soil organic carbon sequestration is 2.3 billion to 5.5 billion tons of carbon dioxide equivalent per year [25].

It is worth noting that D. A. Bossio et al., based on the “nature-based climate solutions” (NbS) proposed by Griscom et al., estimated 12 natural climate mitigation pathways closely related to soil organic carbon and found that the carbon sink potential of 5.5 billion tons of carbon dioxide equivalent per year can be increased by protecting and rebuilding soil organic carbon, accounting for about 25% of the total potential of global natural climate solutions. Cameron Hepburn et al. summarized and estimated the carbon sink potential of 10 pathways on the basis of combing tens of thousands of studies. These include five traditional industrial pathways, including carbon dioxide-derived chemical products, derived fuels, microalgae products, concrete building materials and carbon dioxide flooding oil production, and five unconventional pathways, including biomass energy, rock weathering, forestry technology, soil carbon sequestration and biochar. It is estimated that by 2050, the soil carbon sink potential will be 2.3 billion to 5.3 billion tons of carbon dioxide equivalent/a, of which the available potential mainly comes from the soil carbon sequestration potential of cultivated land and pasture, which is 900 million to 1.9 billion tons of carbon dioxide equivalent/a. Compared with the former study, the latter did not include the biochar potential in the soil carbon sink potential, which was the main reason for the difference in the estimation results.

### 2.3. Soil Carbon Sequestration Management and Market Construction Progress

Farmland soil carbon sequestration is mainly achieved through appropriate farmland management measures. According to the estimation of the United Nations Intergovernmental Panel on Climate Change, reasonable agricultural management measures can increase the global soil carbon pool by 0.4–0.9 PgC/a. If such management continues for 50 years, the global soil carbon pool will increase by 24–43 PgC. Commonly used farmland management measures to increase farmland soil carbon sinks include organic fertilizer, straw returning, no-tillage and fallow tillage. No-tillage can increase the input of labile carbon and reduce the loss of organic carbon caused by soil erosion. Organic fertilizer and straw returning can add organic matter into the soil to increase soil carbon storage. Activities such as no-tillage and cover crops have been shown to promote the increase in soil organic carbon after the implementation of long-term management measures (>10 years). The area of cultivated land in China is 0.79 million mu 191,792 (about 0.19 million hm^2^ 12,786), accounting for about 13.32% of the total land area. Studies have shown that the content of soil organic carbon in China has increased continuously since 1980, indicating that farmland in China has significant potential for carbon sequestration. In addition, China’s carbon emissions trading market has made great progress, but it has not yet been involved in agricultural carbon sequestration trading, especially farmland soil carbon sequestration trading. Internationally, the United States, Australia and other countries mainly carry out farmland soil carbon sink trading practices, generate certified carbon credits through conservation tillage and other farmland management measures, and use carbon offset mechanisms to participate in carbon market trading.

## 3. Literature Review of Forest Carbon Sequestration

Forest ecosystems are the main body of the terrestrial biosphere, storing 90% of plant carbon and 80% of soil carbon in the terrestrial ecosystem. The total carbon storage is twice as much as the carbon content in the atmosphere. The IPCC Second Assessment Report points out that forestry has great potential for carbon sequestration. Between 1995 and 2050, worldwide afforestation and reforestation activities have the potential to sequester 60–87 GtC, equivalent to 12–15% of carbon emissions over the same period. Additionally, forest carbon sequestration has more cost advantages than industrial emission reduction. However, from the perspective of changes in carbon storage, the established forest ecosystem can be either a carbon sink or a carbon source, depending on the dynamic changes in the stock of forest resources. It is estimated that the upper limit of carbon emissions caused by land use change in history is about 200–220 PgC, most of which is caused by deforestation. Assuming that three quarters of the carbon emissions are caused by forest loss and can be restored through reforestation within 100 years, the carbon sink potential of forest ecosystems is about 1.SPgC/a, which can reduce the atmospheric carbon dioxide concentration by 40–70 ppm by 2100. Total deforestation would increase CO_2_ concentrations by 130–290 ppm. Although these are extreme estimates, they illustrate the importance of the way forest resources are used in addressing climate change and sustainable development. The most fundamental thing is to explore, from the perspective of economic growth and institutional change, what type of development mode can make the forest management behavior of society change from destruction and disorderly management to protection, orderly management and sustainable utilization [29,30,31,32,33,34,35].

It should be pointed out that the forest carbon sink service is only one of the many ecological services provided by forest ecosystems, and these services are inseparable in supply. In addition to providing carbon sequestration services, forest ecosystems have diverse functions in maintaining the ecological balance, regulating climate and maintaining biodiversity and hydrological services, which are indispensable for human survival and development. Therefore, forest carbon sequestration is a necessary strategy to deal with climate change with comprehensive benefits and cost advantages.

### 3.1. Estimation of Forest Carbon Storage

Quantifying the role of forests as carbon pools, sources and sinks of carbon emissions and assessing the potential of forest carbon sinks are key issues for terrestrial carbon cycle research and climate change response. Among them, forest biomass is an important indicator to measure and assess the productivity, structure and carbon budget of forest ecosystems. The International Biology Program (IBP) was implemented in the 1960s and 1970s. This program promoted the development of forest biomass surveys and research around the world and provided basic data for the follow-up global research. Since the 1980s, regional and national forest inventory data have provided strong data support for large-scale research, and related research has been widely carried out in this period.

At the global and regional scales, Dixon et al. [35] estimated that the carbon storage of global forest vegetation and soil was 1146 PgC, of which 37% was stored in low-latitude forests, 14% in mid-latitude forests and 49% in high-latitude forests. Additionally, Dixon et al. pointed out that in the 1990s, deforestation in low latitudes resulted in a carbon sink of 1.6 ± 0. At the same time, the increase in forest resources in the middle and high latitudes has achieved a carbon sink of 0.7 ± 0.2 PgC per year, so the total annual net carbon emission of global forests is 0.9 ± 0.4 PgC. Reducing deforestation, increasing afforestation and improving the productive capacity of forest ecosystems through effective management can significantly increase the carbon sink capacity of forests. Based on the DEA-SBM model, Junmin Wei measured and analyzed the forest carbon sequestration efficiency of 30 provinces (cities) in China from 2005 to 2018. With the help of the PSR model, the influencing factors of forest carbon sequestration efficiency were constructed from three perspectives of pressure subsystem, state subsystem and response subsystem, and regression analysis was carried out by using the FGLS model. The results provide a basis for the development of a regional differentiated forest carbon sequestration system. The empirical results show that the average annual carbon sink efficiency of forests in China is only 0.29 [36,37,38,39,40,41].

Specifically, the estimation of China’s forest carbon storage can be divided into three different time periods according to the age of the research object, namely, from 1949 to the end of the 1970s, from the early 1980s to the early 1990s, and from the early 1990s to the 21st century, which in fact correspond to different stages of China’s economic development [42,43,44,45,46,47,48,49,50,51].

In the first period, it is generally believed that the forest biomass carbon pool in the carbon cycle in China from 1949 to the 1970s played the role of a carbon source, but the role of forest carbon sinks from the 1970s to the 1980s is still controversial. Fang used the biomass method based on national forest inventory data and field-measured data.

From 1949 to 1980, China’s total forest carbon emissions were estimated to be 0.68 PgC, and the average annual carbon emissions were 0.022 PgC. Pan suggested that China’s forests became carbon sinks in the early 1970s, not in the late 1970s, and the average annual carbon sink of China’s forests was 0.02 PgC from 1973 to 1981.

In the second period, the large-scale afforestation campaign transformed China’s forests from carbon sources to carbon sinks, and the forest carbon storage increased significantly, but some scholars believe that there are differences between the north and the south. Pan estimated that in the early 1990s, China’s forest carbon pool reached 4.34 PgC, and the annual average carbon sink of China’s forests from 1984 to 1993 was 0.66 PgC/a. It is believed that the significant increase in forest carbon sinks in this period is not only related to the afforestation and reforestation projects in China that started in the 1960s, but also the impact of climate change and the El Nino phenomenon. The age of China’s forests is lower than that of those in the United States and Russia, so the carbon pool is smaller than that of these two countries, but it also shows that there is great potential for development.

In 2005, China’s forest net carbon sink was 97.61 MtC, equivalent to 16.8% of the country’s carbon dioxide emissions.

In the third period, China’s forest carbon storage has further improved due to the extensive development of large-scale key forestry projects. According to Fang, China’s forest biomass carbon pool increased from 4.48 PgC in the late 1970s to 4.75 PgC in 1998, with an average annual cumulative net carbon sink of 0.021 PgC. Fan’s research shows that China’s forest carbon pool has increased from 4.3 PgC to 5.9 PgC in the early 21st century. There are two reasons for this: quantitative expansion and qualitative improvement, as both the forest area and carbon density have increased significantly. Overall, the annual net carbon sink generated by China’s terrestrial vegetation thus far during this period is 0.096–0.106 PgC/a, offsetting 14.6–16.1% of China’s industrial carbon emissions during the same period [52,53,54,55,56,57].

### 3.2. Evaluation of Forest Carbon Sequestration Potential

Compared with the estimation of forest carbon pools in the field of natural sciences, the study of forest carbon sequestration potential has more interdisciplinary characteristics. It is based on the assessment of forest carbon sequestration supply capacity under certain assumptions, which is further based on the biophysical attributes of forest ecosystems and also emphasizes the relationship between these attributes and economic activities. It is necessary to carry out economic analysis on specific forest carbon sink supply mechanisms or activities and their influencing factors.

#### 3.2.1. History of Development

The assessment of global forest carbon sink potential began in the late 1980s, with early studies focusing on the amount of carbon sinks that can be generated by reducing deforestation and increasing afforestation and reforestation activities. Trexler [52] estimated the carbon sink potential for reduced deforestation and forest regeneration in the tropics, and Nilsson [53] estimated the forest carbon sink through reforestation in unforested areas. These two studies show that a total of 700 million hectares of forested land can be used as carbon sinks globally, and that 60–87 GtC will be sequestered through worldwide afforestation and reforestation activities from 1995 to 2050, of which 80% will come from tropical areas, 13% from temperate zones and 3% from cold zones, equivalent to 12–15% of carbon emissions in the same period. Therefore, it is generally advocated to increase the supply of forest carbon sinks through afforestation in the tropics and reforestation and forest management improvement in temperate and frigid zones.

Earlier studies seldom considered the impact of economic, social, environmental and other variables or constraints on forestry activities and their carbon sink potential, as well as the interaction and feedback between these variables. In the following studies, the exchange and integration of economics and natural science have been strengthened, and the neglected problems mentioned above have gradually been included in the scope of the research. From an economic point of view, forest carbon sequestration is a public good, so there will be a problem of insufficient supply. Pigouvian tax, the Coase theorem and public choice theory provide three ways to solve this problem: government, market and autonomous governance. The government’s approach includes subsidies, taxation, property rights allocation, rule-making and other market approaches, mainly carbon emissions trading, and autonomous governance is reflected in the field of climate negotiations. Therefore, the relevant economic research mainly considers the impact of the above factors on the supply potential of forest carbon sinks.

In addition, there are some factors that affect the carbon sequestration potential of forestry. The first is the problem of methodology. Although there are complete carbon sink measurement methods in developed countries, in practice, there exist inaccurate boundaries of measurement and supervision, and in developing countries, due to insufficient investment in national forest resource inventories, there are more errors in the calculation of carbon sinks. The second problem is the additionality and persistence of forest carbon sink projects. The development of forest carbon sinks may reduce incentives for low-carbon technology development in developed countries. These technologies are critical to achieving a climate that is “changing, but stable”.

#### 3.2.2. Forest Ecosystem Carbon Sink Assessment Method [58]

(1) Model construction method

The model construction method is a multi-scale simple calculation model that is not limited to forest aboveground carbon pools and has the function of simulation and prediction based on sufficient basic data according to the types of carbon pool, methodology levels and research areas involved. The representative models include CBM-CFS3, CENTURY, ROTHC, BIOME-BGC, IBIS and CASA. Traditional empirical models, represented by statistical models, estimate the net primary productivity of vegetation using climate-related data, but their application conditions and scope have their own limitations, which cannot reveal the process and mechanism of the plant material production cycle and energy flow. The light use model is a carbon flux simulation model that can achieve scale conversion under the premise of balance, which has the advantage of having less input parameters, but there are some shortcomings in the simulation of soil respiration. The ecophysiological mechanism model based on ecosystem processes and mechanisms can deal with these problems well, comprehensively simulate the response relationship between vegetation photosynthesis, respiration and environmental factors, and simulate and quantify the ecosystem carbon cycle process under past and future climate change conditions. Compared with the traditional empirical model, the most prominent advantages of this model are that it has the function of simulation and prediction and quantifies the environmental impact factors, especially for forest carbon pools with significant growth space. The construction of the model method provides a solid theoretical basis and technical support for coping with environmental changes, formulating scientific environmental protection policies and managing forests empirically. This type of model is mostly based on the parameter data of major stations, meteorological data with daily steps and related ecological and physiological parameter data to study carbon flux. Considering its universality, the calibration and verification of measured data must be carried out for the use and analysis of the model, and the optimization of relevant parameters must be carried out, if necessary, to ensure the application accuracy. However, these global-scale models also share the common shortcoming of extrapolating large-scale ranges based on a limited number of biome data, which ignores potential differences in biome productivity and land use history.

(2) Micrometeorological method

The micrometeorological method can directly measure the dynamic change in the CO_2_ flux, and the results are more accurate by substituting the data on the basis of the established calculation formula. Among them, the eddy correlation method, box method and eddy covariance method are widely used. In the eddy correlation method, based on micrometeorology, the CO_2_ concentration and wind speed and direction are monitored at a standard altitude, and the carbon flux is expressed by the difference between the upward and downward flow of substances in the air through a reference plane [46]. This method can be used to observe and study the forest carbon flux over a long period of time and has high accuracy. In the box method, each part of the vegetation is covered with an airtight box to construct an airtight environment, and then the change in the CO_2_ concentration is recorded over time. The box method can not only measure the CO_2_ flux but also facilitate our understanding of the energy flow of ecosystems [47]. This method has the advantages of a low cost, simple structure and easy operation, but it also has the disadvantages that the method is easily influenced by people, and the box effect and the instrument system error are inevitable. The eddy covariance method is the most direct method to monitor the atmosphere–forest carbon flux. Because of its long observation period, wide range, accurate data and rapid development of technology, it has been widely accepted and recognized by micrometeorology and ecology and is considered to be the standard method to measure ecosystem carbon and water fluxes. Moreover, the data measured by this method are the most authoritative information to test various estimation models. By measuring the wind speed at a certain height and the flow of the measured gas, the method calculates the energy exchange of the interface material, that is, the covariance between the concentration of the material and the vertical velocity. Because its use is not limited by the type of ecosystem and has little disturbance to the ecosystem, it has been widely used in the study of material and energy exchange in various terrestrial ecosystems and the verification of various models. China has carried out multi-directional and all-round interactive verification of forest carbon flux observation at Changbaishan Station, Xishuangbanna Station and Ailaoshan Station. This method needs a reasonable method to interpolate the missing original data of the observation system, and the error of the vertical component in the measurement of the average wind speed is generally corrected by the coordinate rotation method.

(3) Carbon sink remote sensing monitoring method

The progress of spatial information technology has promoted the vigorous development of carbon sink remote sensing monitoring technology, which is driven by remote sensing data, supported by the vegetation or spatial environment database of a geographic information system and coupled with ecological process models to measure carbon sinks, so as to analyze the spatial and temporal distributions and dynamic changes in carbon in forest ecosystems. It can also be used to estimate the carbon stock of large forest ecosystems [51].

Estimation of Carbon Sinks from Optical Remote Sensing Data: Optical remote sensing is a passive remote sensing method, which receives and records the sunlight intensity reflected by ground objects by sensors. There are three common methods to estimate forest aboveground carbon sinks using optical remote sensing data.

Estimation of Carbon Sinks from Microwave Radar Data: Microwave radar is not affected by clouds and weather conditions and is suitable for areas where high-quality optical remote sensing data with a large amount of cloud cover are difficult to obtain. Microwave radar direct measurement directly establishes a regression relationship between the microwave radar data backscatter value or coherence and forest aboveground biomass to estimate forest aboveground biomass and carbon storage; indirect measurement uses microwave radar data to obtain forest structure parameters such as tree height and crown height and establishes a regression relationship between the parameters and forest aboveground biomass for estimation [55]. Microwave radar data analysis requires high analytical skills and professional software, and it is difficult to solve the problem of signal saturation of different degrees.

Estimation of Carbon Sinks from Lidar Data: Based on the principle of laser ranging, lidar has many advantages over conventional methods in the analysis of forest attributes and ecosystem structure. The method comprises the following steps: collecting forest parameter information by setting up a sample plot; calculating the aboveground biomass by using an allometric equation; extracting the forest horizontal structure parameter through the response of ground objects to the spectrum; and, finally, estimating the forest aboveground biomass in the whole study area through regression analysis modeling. Because this method avoids the signal saturation problem of microwave radar data, it can be used to estimate the aboveground carbon storage in tropical rainforests and other areas with a complex forest structure and high biomass [59,60,61,62,63,64,65].

#### 3.2.3. Pay Attention to the Role of Exogenous Water

The external water from silicate rock areas has a strong erosive force. The monitoring results of typical basins show that in the Maocun underground river basin in Guilin, 32% of the exogenous water from the sandstone recharge area in the upper reaches will increase 34% of the carbon sink flux when it enters the karst area in the lower reaches. The monitoring results of the Lijiang River Basin show that when the distribution area of carbonate rocks in a small watershed is approximately 50%, exogenous water has the greatest impact on the karst carbon sink.

### 3.3. Development Status of Forest Carbon Sequestration Market

Forest carbon sequestration trading is an important component of the carbon emissions trading market. The carbon emissions trading market is divided into the mandatory quota trading market and the voluntary emission reduction trading market. This field is an important supplement. Forest carbon sequestration trading belongs to voluntary emission reduction trading, which can be divided into the following categories according to the different certifications or sponsors.

(1) Forest carbon sequestration trading under a national certified voluntary emission reduction mechanism. This is the most important form of forest carbon sequestration trading, which mainly concerns the national or local carbon emissions trading market to offset carbon emissions. In 2011, China approved the pilot work of carbon emissions trading in “two provinces and five cities”, namely, Beijing, Tianjin, Shanghai, Chongqing, Hubei, Guangdong and Shenzhen. The carbon trading pilot market is divided into two types: carbon quota trading and voluntary emission reduction trading. Projects participating in voluntary emission reduction trading shall be registered with the competent national authorities, and the registered emission reductions are called national certified voluntary emission reductions (CCER). Emission control enterprises can either purchase carbon quotas to achieve the compliance target or purchase a certain amount of CCER to offset enterprise emissions. Forest carbon sinks are one of the main types of CCER.A total of 15 CCER projects have been successfully filed, with carbon sequestration afforestation and forest management as the main types of projects.
(2) Forest carbon sequestration trading under a local certified emission reduction mechanism. In addition to state-certified CCER projects, carbon sequestration projects can also apply for local certification, which is currently mainly carried out in several provinces and municipalities that carry out regional carbon emissions trading pilot projects.

The main body of the local carbon emissions trading market is used to offset carbon emissions. The certified emission reductions obtained are generally subject to geographical restrictions and are limited to trading in the region. A few examples include the Beijing Forestry Certified Emission Reduction Project (BCER), Fujian Forestry Certified Emission Reduction Project (FFCER) and Guangdong Provincial Forestry Carbon GSP Certified Emission Reduction Project (PHCER). At present, Beijing has pre-issued 60% of certified emission reductions for the first phase of three projects; Fujian Province has filed seven forest carbon sequestration projects; and Guangdong Province has traded seven provincial forest carbon GSP certified emission reductions. According to statistics, about 350,000 tons of trading volume has been generated.

(3) International certified carbon sink trading. Forest carbon sinks are internationally recognized as high-quality carbon emission reduction products. In addition to participating in the domestic carbon market, the main body of domestic carbon sink development also actively participates in the international carbon market transactions.

Forest carbon sequestration is allowed to be included in the Clean Development Mechanism (CDM), and the first batch of forest carbon sequestration in China is to participate in CDM project trading. At present, the CDM has successfully registered 66 carbon sequestration projects, of which 5 were registered in China, but all before 2011. Since the arrival of the first commitment period of the Kyoto Protocol in 2012, China has not participated in the CDM. At present, some independent carbon emission reduction mechanisms and standards have been established internationally to meet the voluntary emission reduction requirements of organizations and individuals, but some mechanisms and projects can also be used for the performance of the mandatory market, which is basically between the voluntary carbon emission reduction and the mandatory carbon market, including the gold standard (GS) and the international certified carbon emission standard (VCS). There are many restrictions on the GS, and the evaluation process is extremely strict. At present, only three projects in China have been certified by the GS. The VCS is the most widely used measurement standard for the certification of voluntary emission reduction projects around the world. China successfully developed the first project in 2014, and 29 projects have been successfully registered and filed as of July 2021. For example, the Inner Mongolia Forest Industry Group has developed six VCS carbon sinks, with a total transaction volume of 633,000 tons and a transaction value of CNY 9.71 million, averaging CNY 15.3 per ton of carbon sink.

(4) Other forest carbon sequestration transactions. These are mainly aimed at enterprises that fulfill their carbon-neutral social responsibility and voluntarily reduce emissions to enhance their green and low-carbon image. For example, Beijing, Haikou and other places advocate citizens to purchase carbon sinks to fulfill their tree-planting obligations.

These provinces have carried out precise poverty alleviation activities of carbon sequestration per plant, and a variety of forest carbon sequestration projects initiated by the China Green Carbon Sequestration Foundation as the main initiator and promoter have also been partially traded [66,67,68,69,70,71,72,73,74,75].

## 4. Literature Review of Karst Carbon Sink Sequestration

Karst carbon sink refers to the carbon sink effect produced by the process of karstification. Karstification is a process of dissolution and weathering of soluble rocks (mainly carbonate rocks) by water. In chemical terms, water is constantly absorbing the components in the atmosphere. It reacts with CO_2_ in the soil to form carbonic acid, which then dissolves carbonate rocks to form bicarbonate ions and calcium ions. In fact, karstification, like plant photosynthesis, is a reaction process with CO_2_ and H_2_O as the core elements, resulting in a carbon sink effect relative to the atmosphere. However, unlike plant photosynthesis, karstification is an invisible and rapid geological process that occurs over a short time scale, and the migration of carbon is a complex process, where water is a transfer station for carbon. Karstification introduces CO_2_ in the air to the water transfer station, where some of it flows into the ocean with rivers, some is absorbed into the biosphere by trees, some is deposited at the bottom of the water and a small part of it returns to the atmosphere. The investigation of the carbon sink in the Pearl River Basin shows that 4% of the carbon transferred by karstification in the Pearl River Basin is the sediment carbon pool, 27% is absorbed by aquatic trees as organic carbon, 54% is left in the water body to flow into the ocean in the form of inorganic carbon and 15% is returned to the atmosphere due to degasification [76,77,78,79,80,81].

### 4.1. Development Process of Karst Carbon Sinks

China was the first country to carry out karst carbon sink research. In 1995, the International Geoscience Program IGCP379 “Karstification and Carbon Cycle” (1995–1999) led by academician Yuan Daoxian was approved, which marked the beginning of karst carbon cycle research in China. In 1997, it was first proposed that karstification participated in the carbon cycle and had a carbon sink effect around the world. The concept of “karst carbon sink” was formally put forward in 2010. After years of research in the field of karst geology, a major breakthrough was made in the study of karst carbon sinks. The monitoring network of “atmospheric CO_2_-precipitation-vegetation-soil-drip-underground river-sediment” was established, and the Guidelines for Karst Carbon Cycle Investigation and Carbon Sink Effect Assessment (DZ/T 0375—2021) were formed. In 2013, the Fifth Assessment Report of the IPCC Working Group I included “rock weathering carbon sink” and carbonate weathering as aspects of the methods to remove atmospheric CO_2_. Additionally, silicate weathering was listed as one of the methods to remove atmospheric CO_2_. In 2019, four methods to increase karst carbon sinks, including artificial afforestation and grass planting, soil improvement, external water irrigation and aquatic plant cultivation, were actively explored and included in the Annual Report of the Ministry of Ecology and Environment on China’s Policies and Actions to Address Climate Change in 2019. In 2021, the concept of karst carbon sinks was written into the Opinions of the Central Committee of the Communist Party of China and the State Council on Implementing the New Development Concept Completely, Accurately and Comprehensively to Do a Good Job in Carbon Peak and Carbon Neutralization, and the State Council’s Action Plan for Carbon Peak by 2030.

### 4.2. Carbon Sink Capacity of Karst Processes

During the implementation of the IGCP379 project “Karstification and Carbon Cycle” (1995–1999), the preliminary estimation of CO_2_ carbon sinks in karst processes in China and around the world was carried out, in which the carbon sinks in karst processes in China were calculated based on the observation data in fixed sites using the limestone corrosion test piece method, hydrochemistry method and diffusion boundary layer theory. In China, the carbon sink of 70,000 km^2^ of exposed karst area is 1.774 × 107 t CO_2_/a, while the global karst carbon sink is 6.08 × 108 t CO_2_/a, so China’s carbon sink accounts for about 1/3 of the global “missing sink”. This study not only promotes the basic research of karst dynamics but also broadens the field of global change research, which has aroused significant repercussions at home and abroad.

Researcher Liu Zaihua, Institute of Geochemistry, Chinese Academy of Sciences, and Professor Zeng Sibo [81], School of Geographic Sciences, Southwest University, used high-resolution remote sensing and meteorological data as well as a carbonate dissolution balance model to quantitatively study the karst carbon sink flux in carbonate outcrops across the country. The results show that the carbon sink flux is about 6. The total carbon sink produced in karst areas is 17.6 million tons of carbon per year, while the carbon sink can be increased by 38.59 million tons per year by seeding carbonate powder in non-karst areas. Based on the future prediction of eight CMIP6 models, the karst carbon sink flux in China will increase by about 1 ton of carbon per square kilometer per year from 2015 to 2060, and the total carbon sink will increase by about 2.54 million tons of carbon per year (+14.4%), while the potential carbon neutralization potential of broadcast carbonate fines in non-karst areas will increase by about 6.97 million tons of carbon per year (+18.1%).

In the karst area of Southwest China, the environment is a complex structure of carbonate rocks–soils–plants due to the particularity of karst landforms, and plants have been in the situation of CaCO_3_ + H_2_O + CO_2_ ⇌ Ca^2+^ + 2HCO_3_^−^ for a long time. Photosynthetic carbon sinks of plants using CO_2_ through stomata are relatively stable carbon sinks, but in order to cope with the uniqueness of karst environments, plant roots obtain bicarbonate from the soil carbonate system to supplement carbon sources for inorganic carbon assimilation. Whether plants can absorb and utilize bicarbonate is the key point of whether karst carbon sinks can be transformed into plant carbon sinks. Professor Wu Yanyou, Guiyang Institute of Geochemistry, Chinese Academy of Sciences, obtained the most direct evidence of plant utilization of bicarbonate by using two-way isotope labeling technology, proving that karst carbon sinks can be converted into plant carbon sinks. By comparing the absorption and utilization of bicarbonate by different plants, it is proved that karst-suitable plants can regulate the carbon cycle of key karst zones and greatly enhance the carbon sink capacity of ecosystems. Plants can utilize not only the photosynthetic pathway of Rubisco, but also the non-photosynthetic pathway of PEPC to utilize bicarbonate, and the synergistic effect of the two pathways on the absorption and utilization of bicarbonate dominates the transformation of karst carbon sinks into plant carbon sinks. Broussonetia papyrifera experiences more photosynthetic pathway conversion than Morus alba, which further indicates that the karst-adaptive plant Broussonetia papyrifera can efficiently utilize bicarbonate, promote plant growth, enhance the carbon sequestration capacity and more effectively regulate the entire carbon cycle in key karst zones [82,83,84].

### 4.3. Advances in the Study of Increasing Karst Carbon Sinks

In the IPCC-AR5 report, the dissolution and weathering of carbonate rocks are included in one of the four technical methods that can be used to remove atmospheric CO_2_ by human intervention (juxtaposed with terrestrial ecological processes, marine carbon sinks and artificial direct capture). The main methods of artificial intervention to increase karst carbon sinks are vegetation restoration, soil improvement, exogenous water irrigation and the construction of an environment conducive to improving the photosynthetic efficiency of aquatic plants. This has huge potential economic value.

#### 4.3.1. Vegetation Restoration Increases Karst Carbon Sinks

The intensity of karst carbon sinks is different under different land use patterns. In cultivated land or shrub to secondary forest land, the carbon sink produced by karstification can be increased by 5.71~7.02 t/(km^2^·a), and 24.2 t/(km^2^·a) if it evolves to virgin forest land. Roughly 6~26.17 t/(km^2^·a) of vegetation restoration is conducive to the accumulation of soil CO_2_ and the increase in the HCO_3_ flux in spring water. Rocky desertification control can significantly enhance the intensity of karst under the soil. The amount of karst under the soil and carbon sinks in land converted from farmland to forest is about seven times that in cultivated land. After enclosure for more than 20 years, the CO_2_ content in the soil and air increases, karstification is enhanced and the inorganic carbon flux from spring water increases. Vegetation restoration increases karst carbon sinks by 19~23% [85,86].

Land use patterns change the depth of runoff and the concentration of DIC by changing the transpiration, evapotranspiration and concentration of CO_2_ under the soil, and the changes in the depth of runoff and DIC are very different, sometimes even the opposite. The karst carbon sink intensity is the product of the runoff depth and DIC concentration, and the difference in magnitude between them often makes it difficult to evaluate the impact of this land use mode on karst carbon sink intensity, meaning that it is not appropriate to compare them. Zeng [87] used the ratio growth rate of DIC divided by the ratio growth rate of the runoff depth as the impact index of land use change on karst carbon sink intensity at the Shawan Carbon Cycle Monitoring Station in Puding and achieved good results. The ratio was greater than 1, indicating that the increase rate of DIC caused by land use change is higher than the decrease rate of the runoff depth. Additionally, statistics show that that the intensity of karst carbon sinks is enhanced under this condition.

#### 4.3.2. Soil Improvement

Soil improvement refers to the process of taking appropriate physical or chemical measures to improve soil properties, improve soil fertility, increase crop yields and improve the soil environment for human survival, aiming at adverse soil properties and obstacles. Li [88] pointed out that the process of soil improvement is divided into the soil conservation stage and soil improvement stage. The soil conservation stage refers to the adoption of engineering or biological measures to control soil loss within the allowable loss range. If the amount of soil loss is not controlled, soil improvement cannot be carried out. For cultivated soil, farmland capital construction should be carried out first. The purpose of the soil improvement stage is to increase the soil organic matter and nutrient content and improve soil properties and soil fertility. In the long-term comprehensive control of rocky desertification, a series of soil and water conservation technologies in karst areas have been successfully explored, including vegetation restoration projects, terracing projects, land-leveling projects and development projects carried out in the early stage of soil conservation.

Under a series of engineering measures and biological measures such as three-dimensional ecological agriculture, a good rocky desertification control model has been formed, and a set of demonstration models for land consolidation in karst areas has been explored [89,90,91]. Soil improvement work is also being actively carried out, and a variety of successful experiences that can be used for reference have been explored [92]. Pan Yanhua and others took the southeast of Yunnan as the research object and carried out relevant experiments on improving land productivity on lime soil for two consecutive years. The results showed that, compared with traditional cultivation, balanced fertilization, use of water-retaining agents for soil improvement, less tillage and mulching, living mulching, seedling transplanting and reasonable close planting can effectively improve land productivity. They can effectively increase the soil moisture content, improve soil physical and chemical properties and increase the yield and output value [93].

The area of karst rocky desertification farmland in Southwest China is more than 40,000 km^2^. The contents of Ca and Mg are high, and the contents of available trace elements are low. More than 60% of the soils are low-yield soils. The dissolution of carbonate rocks is mainly related to the erosive components in the soil environment, such as CO_2_, water, porosity, organic matter content and pH value. The key to improving karst soil and increasing carbon sinks is to increase the aboveground biomass and soil CO_2_ concentration or moisture to promote the positive occurrence of karstification.

#### 4.3.3. Exogenous Water Irrigation Increases the Intensity of Karst Carbon Sinks

The impact of water on karst carbon sinks is manifested in the water quantity, water quality and movement state, the core of which is the erosion of water. The lower the saturation index of calcite and dolomite in the exogenous water, the stronger the erosion, and the faster the erosion rate of limestone and dolomite in the water. When the external water enters the karst groundwater system, the content of DIC in the water increases continuously, and the carbonate saturation index also increases gradually, from unsaturated to saturated; the karst carbon sink flux can increase by nearly 10 times. The surface water in karst areas is mainly distributed in a few large rivers, which cannot solve the problem of regional water supply. Most of the natural exposed water points belong to seasonal springs, skylights or outlets of underground rivers. Waterlogging occurs in the rainy season and dries up in the dry season, which cannot meet the water demand of residents and agricultural production. This plays an important role in solving the problems of drinking and irrigation for people and livestock in karst areas in the flood and dry seasons by building reservoirs to retain the exogenous water supplied to karst groundwater. CO_2_ is dissolved in the water to produce bicarbonate ions, which can react with Ca^2+^ or Mg^2+^ to precipitate solid carbonate and release water and CO_2_. In northern China, the evaporation and concentration of groundwater with high alkalinity in irrigated farmland lead to the precipitation of CaCO_3_ to form particulate inorganic carbon, which changes the composition of the soil carbon pool and becomes an important source of the soil carbon pool. However, the formation of calcium carbonate deposition will also form new CO_2_, and the microbial activity caused by irrigation will also release CO_2_, which will re-dissolve in the water to dissolve carbonate rocks, resulting in the leaching of dissolved inorganic carbon, which is controlled by vegetation and hydrological conditions. Irrigation increases the annual amount of DIC leaching by nearly 100% in carbonate-developed forest systems [94].

#### 4.3.4. Aquatic Plant Cultivation Enhances the Stability of Karst Carbon Sinks

Aquatic ecosystems play an important role in the carbon cycle of water bodies such as rivers, lakes, reservoirs, wetlands and oceans through biological pumps. Light, temperature and inorganic carbon in the water are important environmental factors affecting the photosynthesis and growth of aquatic plants. The high concentration of dissolved inorganic carbon (DIC) in karst reservoirs has a “fertilizing effect” on the growth of aquatic organisms, which plays an important role in the stability of karst carbon sinks. Endogenous organic carbon produced by DIC is fixed by aquatic photosynthesis in the surface water, which is an important part of the carbon sink of rock weathering. Yang Mingxing used the lipid biomarker method combined with traditional hydrochemical characteristics to calculate the average proportion of endogenous organic carbon to total organic carbon in the Pearl River Basin in the winter and summer. The results showed that the primary productivity caused by the photosynthesis of aquatic plants was relatively strong, and the proportion of endogenous organic carbon and the biomass of aquatic algae were significantly positively correlated with the concentration of DIC. DIC has a fertilization effect on the photosynthesis of aquatic plants.

The spatial and temporal distributions of aquatic plants in the water body are different, and there are dominant species. Therefore, when cultivating aquatic plants to increase the stability of karst carbon sinks, the ecological habits of aquatic plants should be considered. At present, the cultivation of aquatic plants is mainly used for natural landscape beautification, ecological restoration and fishery feed. From the aspect of aquatic plants absorbing dissolved inorganic carbon in the water, the cultivation of submerged macrophytes should be mainly considered for the geological carbon sink function of aquatic plants.

## 5. Main Problems in the Implementation of Carbon Sequestration in the Field of Natural Resources

Since the 18th National Congress of the Communist Party of China, under the scientific guidance of Xi Jinping’s ecological civilization thought, the construction of ecological civilization in China has undergone historic, turning and overall changes from recognition to practice. In the process of practicing the construction of ecological civilization in the field of natural resources, basic systems such as the reform of the property rights system of natural resource assets and the system of natural reserves have been introduced successively. After years of efforts, a unified survey and monitoring system for natural resources in China have been initially established, and the foundation for the development of natural resources has been basically consolidated. The national survey of the status of coral reefs, seagrass beds and salt marsh ecosystems and the pilot survey and assessment of blue carbon reserves such as mangroves, salt marshes and seagrass beds have been completed.

With the advancement of the protection and utilization of natural resources, the level of protection and utilization of natural resources in China has been gradually improved, the supporting capacity of land space has been gradually enhanced and the quality of ecosystems has been continuously improved. According to the results of the third national land survey, the total net increase in forest land, grassland, wetlands and river and lake water surface with strong ecological functions in the past 10 years was 260 million mu. By the end of 2020, the national forest coverage rate reached 23. The forest stock volume has exceeded 17.5 billion cubic meters, and the comprehensive vegetation coverage of grassland has reached 56%. China has restored 1200 km of coastline and 23,000 hectares of coastal wetlands, as well as 9000 mines left over from history, and established more than 1000 hectares of wind prevention and sand control and 1.3 million hectares of rocky desertification control. This will lay a solid foundation for continuously improving the carbon sink capacity of terrestrial and marine ecosystems. Although we have made some achievements, we have also found many problems in the process of collecting and sorting out a large number of documents.

### 5.1. Main Problems in the Development of Forest Carbon Sinks

#### 5.1.1. Policy Implementation Is Difficult and Project Development Is Slow

Since the National Development and Reform Commission suspended the acceptance of applications for voluntary greenhouse gas emission reduction projects, the development of domestic forest carbon sequestration projects has been mainly based on VCS or other regional projects, and the progress has been slow. On the one hand, it is difficult to organize and develop projects. Compared with wind power projects, the development cost of forest carbon sequestration projects is higher, which does not have cost advantages, and the development cycle is longer, which ultimately hinders the sustainable development of the projects. On the other hand, the effective market demand is insufficient. At present, the carbon trading market based on forest carbon sequestration projects is mainly based on voluntary trading, where it is difficult to form an effective demand for carbon sequestration trading; coupled with the long-term periodicity, complexity, ecological externalities and other characteristics of the projects, the economic benefits of project development are not significant in the short term, resulting in constraints on project sustainability.

In addition, up to the suspension of acceptance by the National Development and Reform Commission, the number of forest carbon sequestration projects in China’s voluntary emission reduction trading information platform was relatively small, accounting for only 3.4%, 1.5% and 0.4% of all public projects. At the same time, the proportion of forest carbon sinks in the carbon trading market is relatively small. Among the carbon trading pilot projects in seven provinces in China, the carbon quota is the main content of carbon trading, while forest carbon sequestration trading is a carbon offset project with a small proportion.

#### 5.1.2. The Effectiveness of Project Implementation Fails to Balance Fairness and Efficiency

The poverty reduction attribute is an important part of the multiple benefits of forest carbon sequestration projects. The project developers give excessive preference to the poor groups in the implementation of more professional work such as afforestation and management, such as employing local poor groups to carry out afforestation, forest management and management activities, which, to a certain extent, creates development opportunities for poorer communities and obtains economic income through labor services.

However, due to the relatively weak human capital, lack of professional skills and learning ability of the poor, coupled with the current trend of migrant workers, most of the left-behind villages are an elderly and weak labor force, and their own capacity supply finds it difficult to meet the professional skills needed for forest carbon sequestration project development. As a result, the effect of afforestation or silviculture and management is not good, which is manifested in the low survival rate of forest seedlings after planting, the increase in replanting frequency and area, the further increase in project development cost and the reduction in project operation efficiency. In addition, forest carbon sequestration projects, especially afforestation carbon sequestration projects, mostly carry out afforestation activities in non-forest land or rotation land previously used for grazing and agricultural production, and impose compulsory restrictions on their economic activities, which has a significant impact on the livelihood of surrounding farmers. However, the economic compensation received by such groups in the short term is generally difficult to use to offset the opportunity cost of project development, and if family members do not have a labor force to participate in the project, they may not even be able to obtain any economic compensation, resulting in obvious welfare losses, ultimately leading to challenges to the fairness of project development and damage to social welfare [95].

#### 5.1.3. The Monitoring Mechanism of Carbon Effect Assessment Is Imperfect, and the Regional Differences Are Insufficient

At present, the effectiveness evaluation of forest carbon sequestration projects mainly focuses on the additionality of carbon sequestration, that is, the “carbon sequestration effect assessment” of the project, and the measurement and monitoring methods of forest carbon sequestration have been continuously promoted and developed by the vast number of project practitioners and researchers, mainly focusing on the calculation model and method of carbon dioxide absorption by forests, and the value of forest carbon sinks. Forest carbon sinks absorb carbon dioxide, and industry reduces carbon dioxide in terms of capacity, cost and efficiency differences, taking carbon storage or carbon sinks as the core of ecological service effect evaluation. However, in the short term, compared with carbon trading in conventional energy and industrial sectors, the development of forest carbon sequestration projects often lacks competitive advantages in the carbon sequestration effect, which is why these projects can become an important policy measure for human cooperation to cope with global warming. It mainly benefits from its multiple non-carbon effects of mitigating and adapting to climate change, vegetation restoration and biodiversity conservation, and promoting the sustainable development of communities, which is the key to its position in the whole carbon sink market. In the forest-related provisions of the Paris Agreement reached at the end of 2015, the importance of paying attention to the “non-carbon effect” of forest carbon sequestration projects was also fully emphasized. At present, compared with the increasingly mature research and practice of forest carbon sequestration measurement and monitoring, the exploration of non-carbon effect assessment theory, methods and technical means is still in its infancy, and the relevant assessment and monitoring mechanisms have not yet been established, which, to a large extent, limits the continuous stimulation of market demand for forest carbon sequestration projects.

Suiting measures to local conditions is the key criterion to ensure the long-term sustainable operation of forest carbon sequestration projects. However, at this stage, the development of forest carbon sequestration projects is still based on international standards, which fails to fully consider the differences in and heterogeneity of the specific implementation of these projects. It mainly includes the following two levels.

First, at the regional level of project implementation, the differences are mainly reflected in the property rights of forest land, the scale of forest land/forest circulation, infrastructure construction and physical and geographical characteristics. Since the reform of the collective forest rights system, the ownership and use rights of forests, trees and woodland in China have been clarified, and the enthusiasm of forest farmers to participate in forestry construction has been increasing, which has led to different levels of ownership and circulation of woodland in different implementation areas; the development costs of forest carbon sequestration projects have also been significantly different. In addition, the physical and geographical characteristics and the completeness of infrastructure construction in different regions of China are also different.

Second, at the level of farmers participating in the project, the differences are mainly reflected in the resource endowment. Different family life cycles, different forestry livelihood dependences and different family livelihood decision-making and employment choices lead to significant differences in farmers’ willingness to participate in the project, in participation methods and in participation channels. However, the current forest carbon sequestration project design and implementation are still of a “top-down” type, with farmers’ passive participation and homogenization, without fully considering the differentiated needs of farmers participating in the project, and it is difficult to encourage farmers’ effective participation behavior, which means that the current forest carbon sequestration projects still focus on state-owned forest land, and the coverage of collective forest land is insufficient.

### 5.2. Challenges of Soil Carbon Sinks under the Background of Carbon Neutrality

The great potential and multiple benefits of soil carbon sinks have been recognized globally. Although soil carbon sequestration accounts for only a small part of the international climate action strategy at present, the successful experience of soil organic carbon sequestration projects has provided confidence and support for the formulation of better soil carbon sequestration plans and the promotion of the development of the soil carbon market. In the context of the increasingly urgent requirements of global climate governance, soil carbon sequestration will probably become an important response to mitigate climate change. Nevertheless, it is necessary to recognize the limitations of soil carbon sequestration in practice.

#### 5.2.1. The Research and Technical Support of Soil Carbon Sinks Are Insufficient

As soil is a dynamic and complex system, how to more accurately simulate and reflect the effects of climate change, microbial action and management measures on soil organic matter change and soil carbon sequestration potential, as well as how to achieve large-scale and continuous determination and verification of soil carbon content, is a major scientific challenge. Soil basic information is scattered, unsystematic and incomplete, which greatly restricts the research and practice of soil carbon sequestration in China. In addition, in the practice of soil carbon sequestration, there is a lack of mature and advanced technical support systems for soil sink enhancement, such as the use of straw and other types of agricultural biomass waste carbonization, returning to the field, soil improvement and remediation, and cultivation and management technology improvement, and other advanced human intervention technologies need to be broken through and improved.

#### 5.2.2. The Cost Competitive Advantage of Soil Carbon Sinks Is Uncertain

The immaturity of soil carbon sequestration technology affects the cost–benefit analysis of soil carbon sequestration to a certain extent. Although it can be proved theoretically that soil carbon sequestration has multiple benefits and the cost price may be negative, its value is difficult to realize because its synergistic benefits are difficult to estimate accurately, or because there are externalities. Practical studies show that farmland soil carbon sequestration still faces high costs, which will greatly affect the enthusiasm of agricultural producers and other market investors to participate. At the same time, the current international soil carbon sink market is not active enough and is willing to provide soil carbon.

There are not many enterprises and groups that remit fees. In addition, financial support and incentives are insufficient, even if carbon sequestration compensation fees are paid to farmers in countries with rapid development of soil carbon sequestration, such as the United States and Australia. These fees are also relatively low, and it is difficult to produce greater incentives for agricultural producers to adopt carbon sequestration farming methods.

#### 5.2.3. Soil Carbon Sinks Are Highly Dependent on Continuous Protective Management

Saturation and non-permanence are the relative weaknesses of soil carbon sequestration. Soil carbon sequestration is facing the risk of carbon saturation and returning to the atmosphere. Therefore, it is necessary to maintain the soil organic carbon stock through continuous protective management activities, and improve the mechanism and policy design of carbon sequestration projects to deal with and solve the problem of permanence. This also leads to higher additional management costs in the implementation, trading and supervision of soil carbon sinks. For example, from the application process of the existing soil carbon trading mechanism and action plan, most of the applications are faced with problems such as a cumbersome process, harsh conditions and high environmental protection requirements.

### 5.3. Challenges of Soil Carbon Sinks under the Background of Carbon Neutrality

At present, the mainstream study of the karst carbon cycle with the “rock watershed” as the core is actually the study of the carbon output of the system, ignoring the complex intermediate process, and there are some voices of doubt. Some scholars have proposed that the soil–air CO_2_ consumed by dissolution is less than 1~2% of soil respiration, and karst carbon sinks seem not to be an important “missing sink”. However, some scholars have proposed that carbon sinks in key karst zones calculated based on the dissolved carbon in the outlet of the basin are seriously low, less than 10% of the actual total carbon sink, because most of the carbon input exists in the soil carbon pool in the form of soil organic carbon, and there is also the problem of exogenous acid caused by human activities. These problems are caused by a single or inconsistent research framework. In the global carbon cycle model, the net carbon sink of the terrestrial ecosystem is 2.6 PgC/a, of which the carbon uptake and release of photosynthesis and respiration are 14.1 and 11.6 PgC/a, respectively. In contrast, the global karst carbon sink (0.608~0.89 PgC/a) is indeed much smaller. However, on the one hand, karst carbon sinks are of great significance to solving the problem of the global missing sink (1.4 PgC/a); on the other hand, the current karst carbon sink accounting itself is a part of the global carbon budget accounting, which does not consider the carbon source and carbon sink process of soil–vegetation ecosystems. In addition, the carbon circulation in water systems, aquatic photosynthesis and the catalysis of CA are all rapid carbon turnover processes directly related to biological life activities. The organic carbon in soil, bottom sediment and vegetation undergoes a relatively slow deposition or decomposition process. Even the pathway of transfer from inorganic carbon to organic carbon (“biological carbon pump”) mentioned in this paper has not been directly explained by the buried organic matter itself. One of the key points in proving the stability of karst carbon sinks is to find evidence of the sequestration of modern atmospheric carbon (“new carbon”) in the buried organic matter of karst soils or underwater sediments. It is possible to find direct evidence of atmospheric CO_2_ consumption by the dissolution of modern carbonate rocks in combination with some techniques and methods of organic geochemistry.

## 6. Policy Suggestions

Achieving the goal of “double carbon” is a major national strategy, and karst carbon sequestration, as an important way to achieve “carbon neutrality”, has been included in the top-level design of the national “double carbon action”. Since the 18th National Congress of the Communist Party of China, China has made unprecedented efforts to promote the construction of an ecological civilization, implemented a series of strategies, measures and actions to deal with climate change and participated in global climate governance. Based on the new development stage and the implementation of the new development concept, Guizhou needs multi-party linkage and collective efforts to promote the pilot development of karst carbon sinks in its karst areas. In view of the difficulties and problems in the pilot construction of karst carbon sequestration in our province, we put forward the following suggestions.

### 6.1. Policy Suggestions on Promoting Sustainable Development of Forest Carbon Sequestration

#### 6.1.1. Standards Continuously Expand the Market Share of Forest Carbon Sinks

In order to highlight the responsibility of major powers and enhance the voice of international climate negotiations, we should formulate and promote the voluntary emission reduction of forest carbon in China led by standards and markets, which can not only be in line with international rules but also meet the requirements of sustainable development in the new era, which is efficient, open and transparent, as well as conducive to achieving a win–win situation in addressing climate change and sustainable development. The market demand of multiple subjects should be stimulated for the development of forest carbon sequestration projects, and the share of forest carbon sequestration in China’s carbon emissions trading market should be constantly expanded.

Firstly, we should speed up the development of forest carbon sequestration projects, simplify the application and approval procedures for forest carbon sequestration projects, reduce the implementation costs, reduce the entry threshold for project development and encourage individuals or enterprises to develop forest carbon sink projects with a small scale, outstanding ecological value and good development prospects.

Secondly, we should accelerate the construction of the national carbon emissions trading market, further standardize the contractual management of forests and the clarification and transfer of property rights, explore and promulgate a legal system for the property rights of forest carbon sinks, effectively safeguard the rights and interests of all parties involved in the distribution of carbon sink income and actively guide the formation of a unified national carbon sink trading platform. We should also mobilize the enthusiasm of the public to participate in forest carbon sequestration trading and effectively promote the participation of both supply and demand sides in the forest carbon sequestration market.

Thirdly, we should continue to increase forest carbon sinks, improve the regional forest land transfer system for project implementation and promote the construction of forestry farmers’ organizations such as forestry joint-stock cooperatives, forestry professional cooperatives and forestry technical associations. We should also strengthen the training quality of forest management personnel and the supervision of forest fires and deforestation and strictly prevent the transformation from “carbon sink” to “carbon source”.

Fourthly, we should improve the mechanism of resource integration, promote pilot projects of carbon finance, encourage and guide financial institutions to participate in the carbon sequestration market, enhance the liquidity of carbon sequestration funds, introduce carbon sequestration options, carbon sequestration futures and other related financial derivatives, extend the income chain of forest carbon sequestration projects and encourage more social groups to invest in the carbon sequestration market. We should also fully integrate the resources of ecological construction, agricultural and rural development and other functional departments, strengthen the economic benefits of forest carbon sequestration projects and enhance the comparative advantages of forest carbon sequestration projects. Under the background of the Rural Revitalization Strategy, we should actively integrate forest carbon sequestration into the construction of national key ecological function areas and the implementation plan of the Rural Revitalization Strategy. Based on the abundant forest carbon sequestration project development resources in the region, we should constantly refine policy plans, reach policy consensus and carry out policy pilot projects to improve the local applicability, systematicness and stability of forest carbon sequestration support policies.

#### 6.1.2. Pilot Exploration

The general idea of the implementation of forest carbon sequestration projects has changed from “targeting the poor” to “selectively tilting the vulnerable groups”, focusing on the rigid needs of vulnerable farmers around the project area, which is bound to be a long-term, complex and arduous process through the pilot, promotion and improvement stages.

Firstly, we should give full play to the public service function of the government and combine the National Program for China’s Response to Climate Change and the Action Plan for Forestry Adaptation to Climate Change (2016–2020). The “Work Program for Poverty Alleviation through Ecology” and “Action Plan for Establishing a Market-oriented and Diversified Compensation Mechanism for Ecological Protection” have been implemented, and “Guiding Opinions on the Development of Forest Carbon Sequestration Projects” have been formulated and promulgated to clarify the main objectives, key tasks and guiding policies for talents, prices, investment, finance and market access of the pilot projects, and to give local governments more forest carbon. Local governments are encouraged to actively explore, boldly innovate and be the first to try new approaches. While continuously deepening the practice, the results of policy experiments that have achieved the expected results have been upgraded to supporting policies for forest carbon sequestration projects, and the supporting policy system for forest carbon sequestration has been established and improved.

Secondly, we should also establish a series of preferential and supportive conditions, give market participants more choices in terms of participation mode and depth and constantly explore, summarize and promote project operation modes and benefit-sharing mechanisms with regional characteristics in practice, so as to promote development through policy incentives and benefits rather than compulsory promotion. For example, we should actively support private or non-governmental organizations to apply for approval and registration of forest carbon sequestration projects and give priority and preferential support to the implementation of forest carbon sequestration projects with clear multiple benefit indicators that can be measured, assessed, grasped and supervised. On the basis of meeting the basic land qualification and site conditions, we should weaken the rigid restraint mechanism of carbon trading and forest carbon sequestration project development and moderately relax the constraints of the land selection, scale setting and time limit setting of forest carbon sequestration projects according to local conditions, so as to improve the efficiency of project development. On the basis of developing and expanding all types of carbon funds at all levels in China, such as the China Green Carbon Sequestration Foundation, a special fund for the development of forest carbon sequestration projects with financial support should be added. Combined with the management process of forest carbon sequestration project establishment, approval, registration, implementation monitoring, verification and certification, and CER issuance, we should accelerate the establishment of an assessment and monitoring mechanism that organically combines ex ante, in-process and ex post evaluation of forest carbon sequestration project development and a traceability policy for the simultaneous implementation of third-party assessment.

#### 6.1.3. The Government Promotes the Evaluation of Forest Carbon Sequestration Projects

In the short run, compared with carbon trading in conventional energy and industrial sectors, the development of forest carbon sequestration projects often lacks competitive advantages, but because of its multiple non-carbon effects of mitigating and adapting to climate change, vegetation restoration and biodiversity conservation, and promoting the sustainable development of communities, the development of forest carbon sequestration projects has become the most important aspect. Afforestation and reforestation (AR), reducing emissions from deforestation and forest degradation (REDD) and improved forest management (IFM) have increasingly become important policy measures for human cooperation to cope with global warming, which is the key to their position in the whole carbon sink market. Therefore, it is necessary to speed up the establishment of a quantifiable, comparable and easy-to-operate non-carbon effect evaluation index system for forest carbon sequestration projects, improve the non-carbon effect evaluation mechanism and enhance the attractiveness and competitiveness of the forest carbon sequestration market. One option is to establish the main body of evaluation, in order to construct the selection mechanism of forest carbon sequestration non-carbon effect assessment institutions, which will directly participate in the mid-term and late-stage monitoring and evaluation of forest carbon sequestration projects, so as to identify the third-party assessment institutions and assessors that have an impact on the welfare of project areas, project communities and their farmers as the non-carbon effects of forest carbon sink projects. This includes government agencies at all levels, carbon sink purchasing enterprises and third-party independent institutions dedicated to project evaluation. The second option is to establish and improve the evaluation index system of non-carbon effects of forest carbon sinks. In order to ensure the operability of the assessment, the regions and individuals receiving forest carbon sequestration projects are taken as the target objects of non-carbon effect assessment, including the macro, meso and micro levels of project areas, project communities and farmers, based on the macro, meso and micro levels of forest carbon sequestration project development in the three dimensions of ecology, society and economy. We should construct a quantifiable, comparable and easy-to-operate non-carbon effect assessment index system [24,25]. The third option is to select scientific evaluation methods, taking the region or individual as the basic unit, according to the non-carbon effect evaluation index system, drawing lessons from conditional value, subjective satisfaction and other indicators, directly accessing the subjective perception changes of the evaluation object before and after the implementation of the project, giving each index weight through the combination of subjective and objective evaluation methods, and measuring and comparing the implementation of the project. Then, the effect of the project implementation can be obtained. Based on the multi-period tracking survey method in the early, middle and late stages of project development, a counterfactual model can be constructed to more accurately assess the non-carbon net effect of forest carbon sequestration projects.

### 6.2. Suggestions on Improving Soil Carbon Sink Capacity

#### 6.2.1. Formulate a Soil Carbon Sink to Cope with Climate Change

Despite China’s suspension of joining the “four thousandths” plan, it is still necessary to study and formulate a multi-objective response plan for soil carbon sequestration in order to meet the strategic requirements of “achieving carbon peak by 2030 and carbon neutrality by 2060”, taking into account climate change mitigation, food security and ecological security. It is suggested that soil carbon sequestration, terrestrial vegetation sequestration, marine ecosystem sequestration, geological sequestration and other carbon sequestration pathways, as well as the coordinated management of carbon emission reduction, should be considered from a systematic perspective and concept, and the target of soil carbon sequestration should be set and the implementation approach should be clarified.

#### 6.2.2. Promote Basic Research and Technical Research on Soil Carbon Sequestration

We should strengthen basic research on the impact mechanism of soil carbon sequestration, focus on improving the existing model of soil organic matter turnover processes and gradually establish a systematic research method system for the soil carbon sequestration rate and potential adapted to China’s basic national conditions. We should also promote the establishment of a national soil information service platform, integrate multi-source data from remote sensing, farmland experiments and soil and agricultural management surveys and effectively share basic soil information. We should strengthen research, tackle key problems in soil carbon sequestration technologies, explore and publish recommended technology catalogues for promoting soil sequestration in agricultural production, land consolidation and other fields and guide producers and investors to optimize soil carbon management.

#### 6.2.3. Strengthen Regulation and Management, and Build an Effective Policy System

Firstly, ecological restoration of degraded land should be carried out to enhance the soil carbon sink capacity and promote carbon neutralization. The concept of soil sink enhancement runs through the whole process management of land ecological status investigation and monitoring, ecological restoration planning, ecological restoration compensation, market-oriented mechanism design and ecological restoration project implementation. According to the principles of forest land, grassland and wasteland, we should implement the policy of returning farmland to forests, grasslands and wetlands, scientifically promote the comprehensive control of land degradation and soil erosion, improve soil properties through ecological restoration, increase soil organic matter content and restore and enhance the soil carbon sink potential.

Secondly, the realization of soil carbon sequestration potential depends not only on the development of technology, but also on the support of sound supporting policies. In order to speed up the formation of a unified soil carbon sequestration measurement standard, the measurement of the soil carbon sequestration effect is the key to determining the price of carbon sequestration, but it is difficult to reach a consensus on the existing soil carbon sequestration standards, so it is necessary to speed up the bridging of differences in the understanding of soil carbon sink measurement methods and standards. Secondly, in view of the current low return level of soil carbon sequestration, it is suggested that the government should give appropriate subsidies to soil carbon sequestration and improve the green carbon fund system in China to solve the financing problem of non-market compensation for soil carbon sequestration.

### 6.3. Suggestions on Improving Karst Carbon Sink Capacity

#### 6.3.1. Establish a Framework for the Study of Karst Carbon Cycle

The importance of karst carbon sinks in the global carbon cycle model will be more convincing only if the carbon migration path, interface flux and subsystem occurrence in key karst systems are systematically monitored, simulated and quantitatively calculated, and the interference and influence of human beings in the system are taken into account. Specifically, the study of the karst carbon cycle with the rock watershed system as the core should pay more attention to the vertical atmosphere precipitation on the basis of the horizontally extended closed watershed. The carbon transport process and flux change of vegetation–soil–fracture–bedrock–water, and the mutual restriction and conversion mechanisms of multiple interfaces and multiple scales should be studied. Most obviously, soil–air respiration carbon emission, air–air photosynthesis carbon absorption and soil organic carbon–inorganic carbon conversion should be included in the systematic study and participate in the source and sink calculation. On the basis of the “black box model”, the study of the carbon cycle with karst soil systems as the core should pay attention to the openness, connectivity and particularity of fissures/pipe caves and underground rivers developed in the lower boundary of karst soil.

#### 6.3.2. High-Resolution Monitoring of Biogeochemical Processes

The carbon cycle in key karst zones is a biogeochemical process with coupling of short time scales and long time scales. Biological processes play an important role in the carbon cycle; therefore, the study of the carbon cycle in key karst zones must capture the change process over short time scales to explain the problem. On the basis of identifying the spatial structure and hydrological process of key karst zones, a high-resolution online monitoring system for the subsystems of atmosphere, vegetation, soil, soil water, spring water, cave air, cave drip water and underground river outlets in typical key karst zones should be established by using advanced automatic monitoring instruments. We should also capture the general law of karst carbon transport in the day and night, depending on the season, and the response law to rainfall, climate and human activity events.

## Data Availability

Not applicable.
